# Experiments and Agent Based Models of Zooplankton Movement within Complex Flow Environments

**DOI:** 10.3390/biomimetics5010002

**Published:** 2020-01-05

**Authors:** Mustafa Kemal Ozalp, Laura A. Miller, Thomas Dombrowski, Madeleine Braye, Thomas Dix, Liam Pongracz, Reagan Howell, Daphne Klotsa, Virginia Pasour, Christopher Strickland

**Affiliations:** 1 Department of Biology, University of North Carolina at Chapel Hill, Chapel Hill, NC 27599, USA; mkoz@live.unc.edu (M.K.O.); tomdix@live.unc.edu (T.D.); liamp@live.unc.edu (L.P.); reaganh@live.unc.edu (R.H.); 2 Department of Physics and Astronomy, University of North Carolina at Chapel Hill, Chapel Hill, NC 27599, USA; tdombro@live.unc.edu; 3 Department of Mathematics, University of North Carolina at Chapel Hill, Chapel Hill, NC 27599, USA; maddie15@live.unc.edu; 4 Department of Applied Physical Sciences, University of North Carolina at Chapel Hill, Chapel Hill, NC 27599, USA; dklotsa@email.unc.edu; 5 U. S. Army Research Office, Research Triangle Park, Durham, NC 27709, USA; virginia.b.pasour.civ@mail.mil; 6 Department of Mathematics, University of Tennessee, Knoxville, Knoxville, TN 37996, USA; cstric12@utk.edu

**Keywords:** agent-based model, zooplankton, computational fluid dynamics

## Abstract

The movement of plankton is often dictated by local flow patterns, particularly during storms and in environments with strong flows. Reefs, macrophyte beds, and other immersed structures can provide shelter against washout and drastically alter the distributions of plankton as these structures redirect and slow the flows through them. Advection–diffusion and agent-based models are often used to describe the movement of plankton within marine and fresh water environments and across multiple scales. Experimental validation of such models of plankton movement within complex flow environments is challenging because of the difference in both time and spatial scales. Organisms on the scale of 1 mm or less swim by beating their appendages on the order of 1 Hz and are advected meters to kilometers over days, weeks, and months. One approach to study this challenging multiscale problem is to insert actively moving agents within a background flow field. Open source tools to implement this sort of approach are, however, limited. In this paper, we combine experiments and computational fluid dynamics with a newly developed agent-based modeling platform to quantify plankton movement at the scale of tens of centimeters. We use *Artemia* spp., or brine shrimp, as a model organism given their availability and ease of culturing. The distribution of brine shrimp over time was recorded in a flow tank with simplified physical models of macrophytes. These simplified macrophyte models were 3D-printed arrays of cylinders of varying heights and densities. *Artemia* nauplii were injected within these arrays, and their distributions over time were recorded with video. The detailed three-dimensional flow fields were quantified using computational fluid dynamics and validated experimentally with particle image velocimetry. To better quantify plankton distributions, we developed an agent-based modeling framework, *Planktos*, to simulate the movement of plankton immersed within such flow fields. The spatially and temporally varying *Artemia* distributions were compared across models of varying heights and densities for both the experiments and the agent-based models. The results show that increasing the density of the macrophyte bed drastically increases the average time it takes the plankton to be swept downstream. The height of the macrophyte bed had less of an effect. These effects were easily observed in both experimental studies and in the agent-based simulations.

## 1. Introduction

The term plankton refers to the body of passively floating or weakly motile organisms in a natural water body and is chiefly made up of phytoplankton, zooplankton, bacteria, and viruses, as well as the larvae of many marine invertebrates and fish that inhabit open water. The availability of plankton directly affects the health and existence of fish, shellfish, and larger animals [1,2]. Furthermore, some plankton form blooms, which can lead to increased fouling rates, marine animal mortalities, low dissolved oxygen, and food web disruption [3,4]. Whether plankton are performing a positive or negative role in the food chain, the underlying mechanisms of their movement are poorly understood [5]. Both physical and biological mechanisms affect the movement of planktonic organisms [6,7,8,9], and the dispersal of aquatic organisms can be mostly active, mostly passive, or a mixture of the two [10,11,12].

Mathematical models of plankton movement typically assume that the organisms either: (1) go passively with the flow (similar to tracer particles) [13,14]; or (2) have an additional random motion that can be added to the background flow velocity [15,16] to represent turbulence or small, directed movements. Robust and predictive models of plankton movement are difficult to obtain given their small size (<1 mm) in comparison to the relatively large size of their environments (on the order of meters to thousands of kilometers). Furthermore, it is well known that environmental cues and local structures in the flow environment can drastically alter plankton distributions [17,18], but the incorporation of these high-order effects into mathematical models has been limited [19]. For example, larval and other planktonic organisms can bias their distributions using environmental cues such as light, chemical gradients, gravity, and shear [20,21]. To better understand and predict the movement of plankton, we need to understand how flow and other environmental signals interact with zooplankton behavior to influence their distributions. This will likely involve: (1) determining the level of granularity needed to represent flow fields, such as whether or not smaller scale obstacles such as macrophytes need to be incorporated into flow models; (2) developing computational methods for incorporating zooplankton movement and behavior; and (3) using these methods to determine plankton distributions.

Of particular relevance to this case study is the role that submerged macrophytes play in altering plankton movement at the centimeter scale. Macrophytes provide organisms with shelter from flows across multiple scales, from emergent mangroves on the scale of meters that provide shelter against storm flows greater than 10 m/s to submerged sea grass on the order of centimeters that grow in estuaries and bays with slow flows on the order of 1–10 mm/s [22]. Accurate modeling of flow in vegetative structures (e.g., wetland environments or plant canopies) is necessary to assess the ability of these natural environments to mitigate the effects of storm surge, serve as hosts for biological species, and alter the dispersal patterns of larva [22,23,24,25]. A complete understanding of the fluid behavior within and around these regions is vital when assessing the ability of the vegetated structure to meet predefined goals for their use or consequences associated with their presence in a given environment.

The physical behavior of the flow is altered as fluid passes through the vegetated region since the structure creates resistance, impeding the flow. The presence of vegetation in a flow domain serves to decrease momentum (or inertial effects) of the fluid, increase disturbance to the flow, and possibly change the flow from laminar to turbulent. Vegetative structures also potentially increase the ability of microorganisms to survive within these habitats by preventing wash out. The overall effect of the vegetation on the flow differs depending on several factors, including the density of the bed structure [22,24,26], the height of the plant canopy [24,25], and the heterogeneity of the vegetation within the structure [22,23]. There may also be a significant change in flow behavior above the canopy or vegetated structure, as the physics of the flow in these transition regions are defined, in part, by the resistance to flow offered by the flexible vegetation. These resistance elements create a shear effect on the fluid that encourages the development of vortices. Defining the transition region in these cases may present challenges, as turbulent shear flow also exists in the regions closest to the top of the canopy [25]. Ideally, appropriate models for flow through these systems would consider the distinct multiphysics and multiscale features of the environment. These include descriptions at the blade-scale needed to model the local environments of microorganisms to the macroscale models needed to describe large-scale canopy flows [22].

Simplified analytical models and computational fluid dynamics have previously been used to understand how larger scale vegetation shelters organisms and prevents erosion in strong flows [22,27,28,29]. Similarly, smaller scale plants have an important fluid dynamic role by altering local fluid dynamics, sheltering organisms at the microscale, and altering mixing dynamics in the benthos [30,31,32]. At the canopy scale, a standard approach for modeling fluid momentum within a vegetated region decomposes both the velocity and pressure using time-averaged components and associated deviations [22,33,34,35]. At the smaller scale, detailed spatial and temporal aspects of the flow can be quantified using measurements taken in a flume or by using computational fluid dynamics [36,37,38]. In this paper, we focus our attention on the role of centimeter scale submerged macrophytes in altering flow environments on the order of 1–10 cm/s and the subsequent movement of plankton. We focus on biophysical processes at this scale given its tractability as a first step in developing appropriate flow and agent-based models. We note that the agent-based code developed here can be readily applied across a much wider range of scales if an approximation of the flow field is available as an input.

We use *Artemia* spp., a genus of saltwater plankton commonly known as brine shrimp, to experimentally quantify how the presence, height, and density of simplified macrophytes alter their distributions in flow. Brine shrimp, also known as sea monkeys, serve as an excellent model organism given their availability and ease of culturing [39]. Experimentally derived “diffusion” coefficients describing the random active movements of newly hatched nauplii have been determined previously by Kohler et al. [39]. This organismal system can also be used to develop behavioral models such as movement towards light or a concentration gradient. Behavioral models can then be incorporated into flow models to determine the impact of behavior on distributions and dispersal rates. As a first attempt to mathematically describe and experimentally quantify the movement of plankton in sheltering structures, we injected newly hatched nauplii into physical models of macrophyte beds that have been immersed in a flow tank. The concentrations of brine shrimp were tracked over time in two regions downstream of the physical model. The flows within and around the physical model were determined numerically and validated experimentally using particle image velocimetry.

As we resolved the fluid dynamic models of individual plankton and their basic locomotory characteristics, we then used these results to specify agent-based models in order to explore emergent patterns or behaviors in the population as a whole. Agent-based models (or individual-based models) are models in which individuals are described as autonomous entities which can then interact with each other and/or their environment. They have adaptive behavior which can include continual adjustments to changes in their environment, the presence and behavior of other agents, or to their own internal states [40]. These models are typically stochastic in nature and are particularly well suited to the task of exploring how simple, single-organism dynamics can give rise to population-level, aggregate phenomena. For this case study, we developed an agent-based model that assumes the brine shrimp move at the local fluid velocity and with some additional random motion representing active swimming. The distributions of brine shrimp over time predicted by the agent-based model are then reported.

The use of this modeling framework and associated software released with this paper extends well beyond the case study considered here. Agent-based models enjoy widespread use across a variety of ecological contexts with varying levels of complexity (e.g., [41,42,43,44] or see [45] for a long list of citations). However, from a mathematical perspective, parsimony is critical in forming an agent-based model that can inform theory. When properly formulated in the context of lab or field observations, they can be powerful tools for forging a connection between key individual-level behaviors and macroscopic group properties [46,47]. This understanding can then be used to form robust mathematical characterizations of population movement.

While a variety of software packages exist that provide comprehensive simulation environments for agent-based modeling [48], they tend to be for high-level, general use, and are not well suited to the specific challenges of incorporating fluid–structure interaction data for swimmers in continuous 2D or 3D environments. *Planktos* is an object-oriented modeling environment capable of interfacing with 2D or 3D flow data. Custom agent behavior and response to flow gradients can be tested and visualized both quickly and easily, with 1D analytical flow fields available internally for environmental comparison. *Planktos* is documented for additional use by other researchers and has been made permanently available as open source software on GitHub.

## 2. Methods

Here, we provide a brief overview of the experimental and theoretical frameworks used to describe the movement of zooplankton in a complex flow environment. Newly hatched *Artemia* nauplii were injected into physical models of macrophyte beds in a flow tank. The distributions of nauplii over time were tracked at two locations downstream of the physical model. The flow fields were reproduced using computational fluid dynamics and validated experimentally. Agent-based models were used to describe the distributions of nauplii as they were advected with the fluid and moved with an additional random motion that models active swimming. The macrophyte density and height were varied for both the experiments and the simulations.

### 2.1. Relevant Dimensionless Numbers

There are a few dimensionless numbers that are useful when analyzing the movement of plankton. The Reynolds number Re is defined as

$$Re=\frac{\rho UL}{\mu }=\frac{UL}{\nu },$$

where ρ is the density of the fluid, μ is the dynamic viscosity, *U* is a characteristic velocity (such as the swimming speed), *L* is a characteristic length (such as the body length), and ν=μ/ρ is the kinematic fluid viscosity. The Reynolds number can be thought of as the ratio of inertial forces to viscous forces acting in the fluid flow. An example of biological flows where Re>> 1 can be seen in the case of fish swimming, while the movement of bacteria by flagella occurs at Re<< 1. Low Re flows such as those experienced by bacteria are reversible, which means that if you drive motion with a boundary then move that boundary back through the same path, the fluid will return to its initial state [49]. During ontogeny, *Artemia* grow across a range of body sizes from hundreds of microns to 1 cm. Newly hatched larva, or nauplii, swim with their two antennae in a motion that is similar to a breaststroke near a Reynolds number of 1, whereas adults row with a series of swimming appendages near a Reynolds number of 40 [50,51]. In this study, we used nauplii that were within a few days of hatching such that they swim near Re=1.

Another useful dimensionless number is the velocity ratio, $V_{r}$, that describes the ability of an organism to swim against a flow and is useful when considering the relative importance of active and passive transport and determining which models of dispersal are appropriate [19]. This ratio is given as

(1) $$V_{r}=\frac{u_{s}}{U_{v,h}},$$

where $u_{s}$ is the organism’s swimming velocity and $U_{v,h}$ gives the vertical and horizontal velocity components of the background flow, respectively. For larval planktonic organisms, Re and $V_{r}$ are typically on the order of one or less. For larger and/or adult organisms, the interaction between active locomotion and physical transport become more complex. For low values of $V_{r}$, passive drifting is the predominant mechanism of dispersal, and organisms are swept along with the background flow. For intermediate and high values of $V_{r}$, self-propulsion becomes significant and behavior can greatly influence movement and resulting dispersal patterns. For *Artemia*, the velocity ratio, $V_{f}$, varies from 0.001 to 100, which is calculated using swimming speeds that vary from about 1–10 mm/s with background flow speeds that range 1–1000 mm/s. These parameters represent conditions ranging from stagnant regions of salt lakes to typical flows plankton would experience in the ocean. In the experiments described here, $V_{r}$ ranged from about 0.02 to 10, motivating our agent-based model that considers both advection and diffusion.

### 2.2. Macrophyte Models

Submerged macrophyte beds were approximated as rigid cylinders arranged in a regular grid and 3D printed for flow tank experiments. For all models, the base consisted of a 7.5 cm × 15 cm × 0.25 cm rectangular prism, bonded to rigid cylinders that were 0.25 cm in diameter. To vary the density of the macrophyte bed, models were created that contained evenly spaced cylinders in 8 × 15, 10 × 20, and 15 × 30 arrangements. Note that the longest dimension was in the direction of flow. In addition, models of each density were constructed with uniform cylinder heights of 1, 2, or 3 cm for a total of nine models. The models were designed as one component in Autodesk Fusion 360 and saved in stl format. Using stl files, models were 3D printed on uPrint SE Plus, using ABS as build material, at the UNC Makerspace. The models used for particle image velocimetry were lightly spray painted black.

### 2.3. Flow Tank Set Up

**Figure 1:**
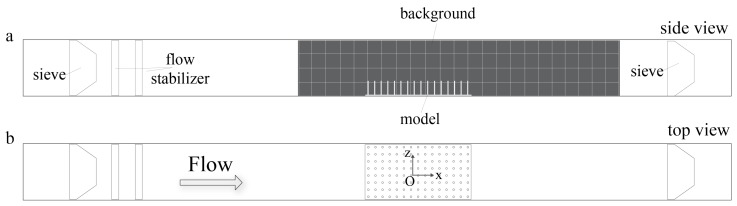
Experimental set up of submerged macrophyte models in the flow tank: (**a**) the view of flow tank setup from side; and (**b**) the view of flow tank from above. Flow tank is 100 cm (L) × 8 cm (W) × 8 cm (H).

For the flow experiments, the physical models of submerged macrophyte beds were placed at the center of the bottom of an optically accessible 8 cm × 8 cm × 100 cm plexiglass flow tank that was custom built based upon Vogel and LaBarbera [52] (see Figure 1). The models were held in place using marine putty as if submerged and rooted. Two *Artemia* sieves were placed at far upstream and downstream from the model in order to prevent feeding the injected organisms back into the system. The dimensions of the sieves were 8.64 cm × 7.87 cm × 4.45 cm with 180 micron mesh size. A black background with thickness less than a millimeter was placed in the flow tank to enhance the contrast between the *Artemia* nauplii and the background. The background included a grid with 2 cm × 2 cm squares to facilitate tracking. Constant flow was generated by a propeller attached to a variable speed motor, and the flow within the empty channel had a maximum average velocity of 4.4 cm/s. The flow tank was filled with seawater of 35 ppt salinity, and the height of the water was kept at 7.5 cm to prevent overflowing when the propeller was in action.

### 2.4. Measurements of Flow

The temperature of the salt water in the flow tank was about 20 ${}^{\circ }$C such that its density was assumed to be ρ = 1025 kg/m${}^{3}$ with a dynamic viscosity μ = 0.00108 Ns/m${}^{2}$. A parabolic profile was used to set the inflow conditions for the computations, and the volumetric flow rate was matched to that measured experimentally for the case of no model. This resulted in a maximum inflow velocity of 0.0444 m/s. Setting the characteristic velocity to this maximum value, *U* = 0.0444 m/s, one can calculate Re at the scale of the individual cylinders and at the scale of the macrophyte bed. Using a characteristic length equal to the height of the cylinders, *h* = 0.01–0.03 m, the bed-based Re ranged from 421–1264. Using the diameter of the cylinders, *D* = 0.0025 m, the cylinder-based Re was about 105. This range of Re is reasonable for using direct numerical simulation of the Navier–Stokes equations to obtain the flows around and over the macrophyte beds.

#### 2.4.1. Particle Image Velocimetry

The fluid velocities within the flow tank were obtained using 2D particle image velocimetry (PIV) and used for validation of the numerical simulations. PIV is a non-intrusive technique used to obtain instantaneous and spatially resolved information on a flow field by recording the single or multiple exposed images of passive tracer particles suspended in the fluid. In general, a monochromatic coherent light source is used to illuminate these small seeding particles, and the positions of the particles are recorded at set time intervals. The displacement (Δx,Δy) of a single particle or group of particles is deduced from correlation-based post processing of these images. A map of the velocity of motion of the tracers in the flow field can thus be obtained using (Δx/Δt,Δy/Δt). For more information on this technique, please refer to Raffel et al. [53] and Willert and Gharib [54].

In this work, the laser sheet was generated by a 1 W Nd:YAG, Diode Pumped Solid State Laser (DPSS) laser manufactured by LaVision GmbH, which emits light at a wavelength of 532 nm with a maximum modulation of 10 kHz. The laser beam was converted into a planar sheet approximately 3 mm thick using a set of focusing optics. Uniform seeding of the fluid was accomplished by inserting 10 micron glass hollow spheres (LaVision GmbH) into the flow tank and mixing to achieve a near homogeneous distribution prior to each experiment. A Photron FASTCAM SA3 high-speed camera with Tokina Macro 100 F2.8 D lens attached was used for capturing images with a 1024 × 1024 resolution at 500 Hz for 1 s. The spatial resolution was 0.017 mm per pixel. All frames were pre-processed in order to remove glares and unwanted brightness in images. To do this, local minimum of series were subtracted from each frame. Then, the cylinder array and regions outside the flow were masked before analyzing images in Davis 8.0 (LaVision). Velocity vectors were calculated using cross-correlation, multi-pass iterations with 2 passes. The size of the interrogation windows was set to 64 × 64 and 32 × 32 pixels with 50% overlap, respectively. The resulting flow fields were post-processed using a non-linear filter to remove faulty vectors. Finally, the velocity fields of the entire image set were averaged to get the average velocity profiles.

**Figure 2:**
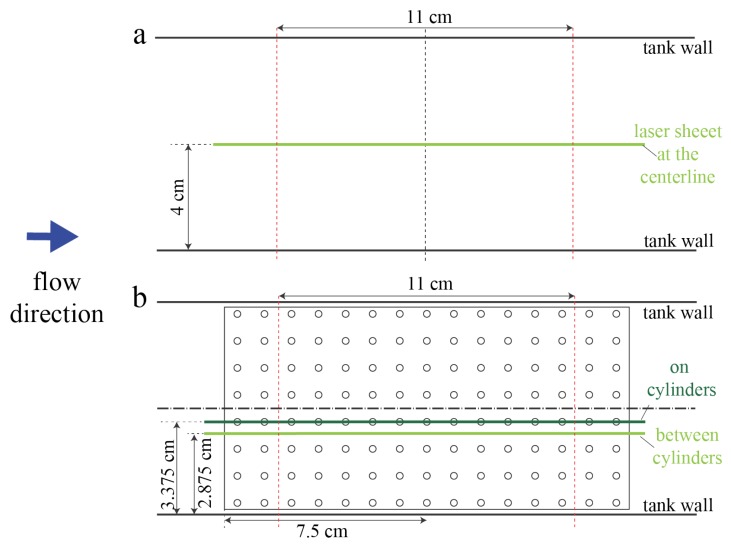
Position of laser sheet for the empty tank (**a**) and the cylinder models (**b**). Light and dark green lines denote the position of the laser at different times. The vertical dashed-dotted line is the y-axis. Dashed-dotted horizontal lines represent the x-axis for each setup. Red dashed-lines denote the beginning and the end the region that the camera can capture for PIV. The dark blue arrow shows the direction of flow during PIV measurements.

For validation of numerical simulations, PIV measurements were taken in an empty tank and in the tank with the 10 × 20 2-cm macrophyte model. Flow velocities within two different planes tangential to the flow were measured. The first plane passed between the middle two cylinders rows. The second plane passed through the center of the cylinders in the middle of the model. Figure 2 shows the position of laser sheet for these two scenarios.

Note that it was difficult to resolve the flow field within the model due to the narrow gap between cylinders and the fact that the model is optically inaccessible. However, PIV measurements still produced sufficiently detailed information about the flow field for comparison with numerical simulations since we compared the regions that were optically accessible.

#### 2.4.2. Computational Fluid Dynamics

**Figure 3:**
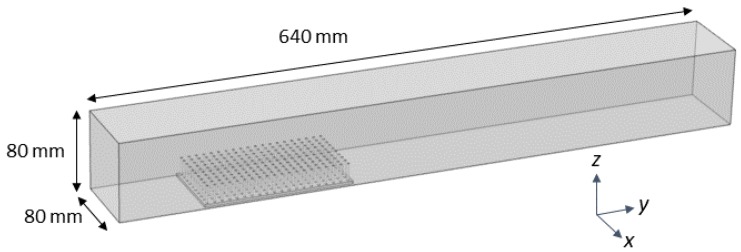
Computational domain for COMSOL fluid physics simulations. The domain represents the experimental setup in the flow tank and is an 80 mm × 640 mm × 80 mm box. Parabolic inflow conditions and zero pressure outflow conditions are used. No slip conditions were used elsewhere.

COMSOL Multiphysics 4.5 was used to mesh the fluid domain and solve the steady Navier-Stokes equations. The single-phase laminar flow physics solver was used to perform a stationary study (e.g., ∂u/∂t=0). The fluid domain had dimensions of 80 mm × 320 mm × 80 mm, similar to the actual flow tank. The density and dynamic viscosity of the fluid was set to ρ = 1025 kg/m${}^{3}$ with a dynamic viscosity μ = 0.00108, within the range of typical seawater. The entrance of the domain was given an inlet boundary condition of parabolic flow with a maximum velocity of 44.4 mm/s in the center of the domain, unless otherwise noted. The back wall was given a zero pressure outlet condition. All other walls used a no-slip boundary condition. A free tetrahedral mesh of normal resolution calibrated for fluid dynamics was used to discretize the fluid domain (see Figure 3).

The same .stl files used for 3D printing were used for the COMSOL simulations. These models were positioned at the bottom of the computational flow tank and in the middle of the domain. The steady Navier–Stokes equations were solved in the rectangular domain with the model region subtracted. No slip conditions were employed on the model boundaries and sides of the domain. Note that, for the simulations without a model, the plate without cylinders was positioned in the bottom of the tank.

### 2.5. Dispersal Experiments

The concentration of plankton under steady background flow in the presence and absence of macrophyte models was recorded at spatial locations downstream of the models. Experiments were repeated three times for each model (including the no model case), for a total of 30 experiments. The video recordings were processed to quantify dispersal patterns as a function of macrophyte density and height. For each scenario, the numbers of plankton measured in each experiment were added together to obtain a combined distribution.

#### 2.5.1. Preparation of *Artemia* Larvae

One gram of *Artemia* cysts (Grade A Brine Shrimp Eggs, Brine Shrimp Direct) was hatched in 1 L of seawater with a concentration of 35 ppt. After 48 h, nauplii were separated from empty shells by filtering the hatching water using a 180-micron *Artemia* sieve. The nauplii were transferred into a 2-L beaker filled with fresh seawater at 35 ppt. Note that the filtering process did not completely separate empty shells, and negligible amounts of shells and unhatched eggs were present in the final mixture.

#### 2.5.2. Injection of Brine Shrimp into the Flow Tank

Before each experiment, 0.5 L of salt water with *Artemia* was filtered and concentrated into a 40-mL solution. As a result of the concentration process, organisms were introduced into the flow as a dense patch with minimal flow disturbance. After each experiment, unused *Artemia* were returned into the 2-L beaker and given sufficient time (≈10 min) to recover. This procedure was put in place to ensure that the nauplii were active during the experiments.

**Figure 4:**
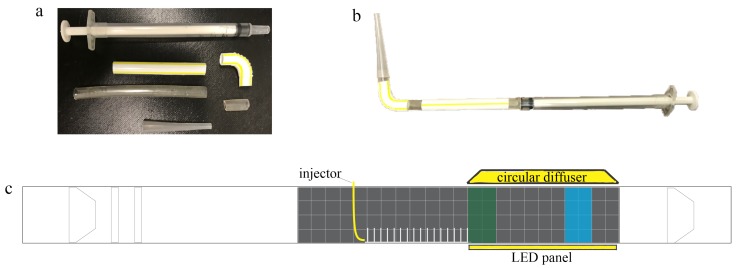
Larvae injector with a modified tip design: (**a**) the injector was constructed using a 1-mL insulin injector, 65-mm and 10-mm sections of aquarium tubing with 5 mm diameter, a straight 45-mm plastic straw piece, a 19-mm bendy section of the straw, and the tip of a 3-mL pipette that has an opening that is 2 mm in diameter and 35 mm in length; (**b**) the complete injector as assembled; and (**c**) flow tank setup for the dispersal experiments. The injector was positioned immediately upstream of the macrophyte model. Green and blue rectangles denote the green and blue zones, respectively, where the concentrations of brine shrimp were recorded. A circular diffuser and LED panel wrapped in plain paper to serve as a diffuser were placed above and below the tank immediately after the model.

Larvae were placed in the flow tank using a 1 mL injector with a modified tip, as shown in Figure 4a,b. The injector was designed to minimize flow disturbance when submerged in the tank. Before each injection, 0.1 mL of air was pipetted before taking up 0.2 mL of the nauplii and salt water. This initial bubble was used to signal when the injection was complete. Finally, 0.1 mL of air was pipetted to serve as a seal to prevent the early release of nauplii into the flow. Between each experiment, the mouth of the injector was gently washed with salt water to remove any remaining larvae or eggs. All injections were performed at the center of the flow tank in the region directly upstream of the model and above the base plate, as shown in Figure 4c. The average injection speed was 33.31 mm/s with 11.89 mm/s standard deviation and 32.97 mm/s median for 30 trials.

#### 2.5.3. Recording Dispersal of Plankton

Brine shrimp nauplii were recorded using three Sony Alpha 6300 cameras with 16–50 mm stock lenses. One camera was used to record the injection of larvae and their initial movement through the macrophyte model. The concentration of brine shrimp directly downstream of the macrophyte model was recorded using the remaining two cameras. One camera was placed immediately after the model to record the nauplii within a region named the “green zone”, and the other camera was placed 15 cm downstream of the model, and this region was named the “blue zone”. Each zone consists of eight 2 cm × 2 cm cells, making each zone 4 cm (W) × 8 cm (H). All recordings were taken at 30 fps with 4K resolution. The shutter speed was set to 1/640 s.

To resolve individual brine shrimp in a relatively large volume of fluid, significant attention was given to lighting. One Flashpoint FPLCL1144R LED Circular Diffuser and one Fositan TL-336AS LED Video Light with custom diffuser were used to evenly illuminate the volume of water in the flow tank. The circular diffuser was set to maximum intensity and placed above the flow tank right after the model. The Fositan LED panel was also set to maximum intensity and placed below the flow tank. A plain white piece of paper was used to cover the Fositan LED panel to act as a diffuser.

#### 2.5.4. Processing Recordings

Recordings from the camera positioned upstream of the macrophyte model were used to determine the injection velocity of the brine shrimp solution. In this case, it was assumed that the brine shrimp act as passive tracers that move at the local fluid velocity. The recordings from the cameras placed downstream were then used to determine the number of nauplii over time in the green and blue zones. Videos were played frame by frame using Quicktime Player 7.0. Each zone consisted of eight 2 cm × 2 cm squares. The organisms in each square were counted at 0.5 s intervals and recorded into an Excel spreadsheet for further processing. To improve the precision of counting the nauplii, a screenshot of the region of interest was taken and each organism was marked as it was counted using Adobe Photoshop CC (Adobe Photoshop CC Version 20.0.6, Adobe Creative Cloud).

The number of brine shrimp in each zone and for each increment in time for the three experiments were combined for comparisons between model cases and the agent-based simulations. All experimental results were plotted in MATLAB [55].

### 2.6. Agent-Based Modeling (ABM) Framework: *Planktos*

For this project, we make use of an in-house, open source, agent-based framework called *Planktos* [56] that is capable of exploring emergent patterns of organisms in complex flow regimes based on individual-level behavioral rules. Agent-based models (or individual-based models) are models in which individuals are described as autonomous entities which can then interact with each other and/or their environment. In this setting, simulated organisms can have adaptive behavior which can include continual adjustments in response to changes in their environment, the presence and behavior of other agents, or to their own internal states [40].

For this preliminary study, brine shrimp were assumed to move via a random walk such that their effective diffusivity was comparable to experimental data from Kohler et al. [39] and their mean drift corresponded to the local flow velocity. Numerically speaking, this means that the location of each individual agent (brine shrimp) was independently updated at each time step using the time-invariant flow field simulated in COMSOL (for a given model geometry) and a three-dimensional normal distribution as given in the equation

$$x_{t+\Delta t}=x_{t}+\varepsilon \left(x_{t}\right),\;\;\;\;\;\;\mathrm{where}\;\;\varepsilon ∼N\left(v\left(x_{t}\right)\Delta t,\Sigma \Delta t\right)$$

with Σ given to be the 3 × 3 covariance matrix with the variance as derived from Kohler et al. [39] along the diagonal and zeros elsewhere, and $v(x_{t})$ the fluid velocity at $x_{t}$, found by linearly interpolating the numerical flow field grid specified in COMSOL. In the case that agents crossed a domain boundary corresponding to the tank wall or the surface of the fluid, they were placed on the boundary by translating back to the nearest point on the edge of the domain. If agents crossed the upstream or downstream boundary of the domain, they were considered to have left the experimental environment and were no longer tracked in any way for the remainder of the simulation. In this first study, individuals did not interact with each other and they did not modify the flow field.

While various software packages exist that provide comprehensive simulation environments for agent-based modeling [48], they tend to be for high-level, general use and are not well suited to the specific challenges of incorporating fluid–structure interaction data for swimmers in continuous 2D or 3D environments. *Planktos* is an object-oriented modeling environment written in Python and specifically built for interfacing with numerical 2D or 3D flow data, including time-varying flows (although this feature was not used in this study). Custom agent behavior and response to flow gradients can be tested and visualized both quickly and easily, with 1D analytical flow fields available internally for environmental comparison [56].

Some of the capabilities of *Planktos* that we specifically used in our study of *Artemia* include:

- VTK-based import of 2D or 3D time-dependent fluid flow data and flow interpolation between grid points
- Fluid flow tiling with periodic boundary conditions to expand the domain
- Selection of boundary conditions for agents
- 2D and 3D plotting of agent movement, including summary statistics and density histograms

### 2.7. Agent-Based Simulation and Experimental Results

There are several challenges with directly comparing the experimental results with the agent-based simulations. It is difficult to track individual *Artemia* nauplii (≈1 mm) as they move through a relatively large 3D volume of fluid (≈4000 cm${}^{3}$). We could not track individuals throughout the duration of the experiment, but we could count the number of nauplii present in each zone in 0.5 s intervals. On the other hand, we could easily track individuals in the agent-based simulations. Because we knew the location of each individual at any time, we could calculate the exact times that each individual arrived in the green and blue zones (the arrival times). In the experiments, we could only report how many individuals were present in the zone at each time, and these individuals could remain in the zone for several time increments. Furthermore, the amount of time individuals spent in the zone was proportional to the density of cylinders in the macrophyte models, as this slowed the local flow velocities. We report arrival times for the agent-based simulations and the number of nauplii in each zone at each time increment for the experiments. Despite the differences in the data we could obtain from simulations and experiments, both metrics do reveal similar trends.

There are also some differences in parameter values describing the experiment and used in the simulations. For example, we do not know the number of brine shrimp injected into the flow tank experiments, which likely varied to some degree between experiments. For consistency, the agent-based simulations included 10,000 agents in each simulation. We estimate that each experiment included a couple thousand brine shrimp. We also do not know the effective diffusivity of the brine shrimp in each experiment. The nauplii were approximately 48 h post fertilization, but there was some variation in incubation time and activity due to the time of day and treatment of the brine shrimp. In the agent-based simulations, we always used the same diffusivity reported by Kohler et al., but, for newly hatched nauplii, potentially of a different species and ordered from a different supplier. Finally, there were some variations in the volumetric flow rate of the experiments. The control dial was always set to the same setting, but the presence of models as well as their height and density changed the resistance to flow and the resulting flow speed. In the simulations, the peak inflow velocity was always set to 44 mm/s. For these reasons, we do not directly compare experiments and simulations, but look for similar trends in dispersal patterns.

## 3. Results

### 3.1. Flow Fields

#### 3.1.1. Validation of COMSOL Results

**Figure 5:**
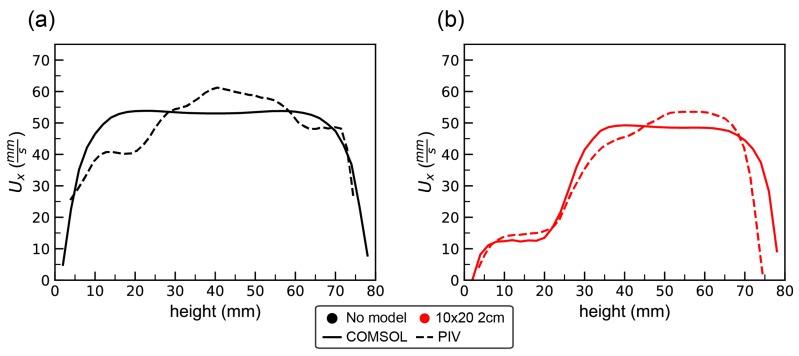
The *x*-component of the velocity as a function of height taken along a vertical line positioned in the middle of the model for the COMSOL simulation (solid line) and experiment (dashed line): (**a**) the flow profiles with no model; and (**b**) the profiles with the 10 × 20 2-cm model.

Particle image velocimetry was used to determine the flow field in the empty tank and around each of the models. The volumetric flux was used to set the appropriate boundary conditions for the COMSOL simulations. For the no model case, the parabolic inflow was used with a maximum velocity of 44 mm/s. For the 10 × 20 2-cm model, the maximum inflow velocity was set to 31.3 mm/s, as determined from calculating the volumetric flux from PIV data. Note that there was some variation in the volumetric flux between experiments due to the increased resistance to flow with larger models and due to variations in the motor output. Figure 5 shows the *x*-component of the velocity (in the direction of flow) as a function of height taken along a vertical line placed in the center of the model for the simulations (solid line) and experiments (dashed line). Figure 5a shows the results for the no model case. The simulations exhibit smooth flow typical of what would be expected in a channel for $Re\approx 10^{2}$–$10^{3}$. Similar flows were measured in the flow tank with some variations as a function of height. This is likely due to the action of the motor, the air–water interface at the top of the tank, etc. Figure 5b shows the velocity profile for the 10 × 20 2-cm model case. Both the simulation and experiment for the 10 × 20 2-cm model reveal considerably slower flows within the model with a rapid increase in velocity above the model.

#### 3.1.2. Flow Dependence on Cylinder Density and Height

**Figure 6:**
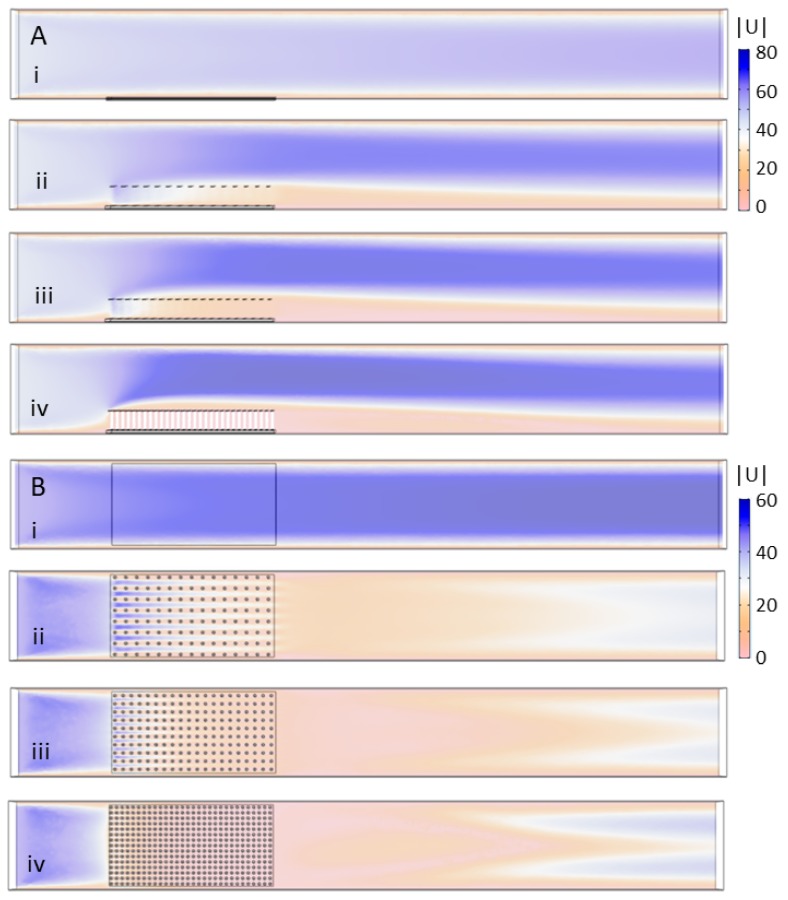
Velocity magnitude of the flow through models of varying cylinder density: (**A**) the velocity magnitude in mm/s taken in a vertical plane tangential to the direction of flow and through the center of the tank; and (**B**) the velocity magnitude in a horizontal plane 1 cm above the bottom of the tank. The models considered include: (i) no model; (ii) 8 × 15 cylinders; (iii) 10 × 20 cylinders; and (iv) 15 × 30 cylinders.

In this section, the maximum inflow velocity was set to 44 mm/s for all cases, and the results were used to generate a consistent flow field for the ABM simulations. Note that the stationary equations of fluid motion were solved to make both the CFD and the ABM simulations tractable. Figure 6 shows the velocity magnitude for the case of no model (i) and the 2 cm models with spacing set to: (ii) 8 × 15; (iii) 10 × 20; and (iv) 15 × 30. Figure 6A shows the side view of the flow field where the velocity magnitude, |U| in mm/s, is displayed in a vertical plane that cuts through the center of the flow tank. Figure 6B shows the velocity magnitude in a horizontal plane taken 1 cm above the bottom of the tank. By comparing (i)–(iv), it is clear that increasing cylinder density has a strong effect on the magnitude of the flow between the cylinders. For the 8 × 15 (ii) and 10 × 20 (iii) models, there is some non-negligible flow through the first several rows of cylinders with little flow through the downstream half of the model. For the densest 15 × 30 model (iv), there is little flow through any of the rows of cylinders. The flow is also directed upwards such that there is a sheltered region extending about a half centimeter above the model. For all densities ((ii)–(iv)), a relatively long wake with slow flow is observed downstream of the models.

**Figure 7:**
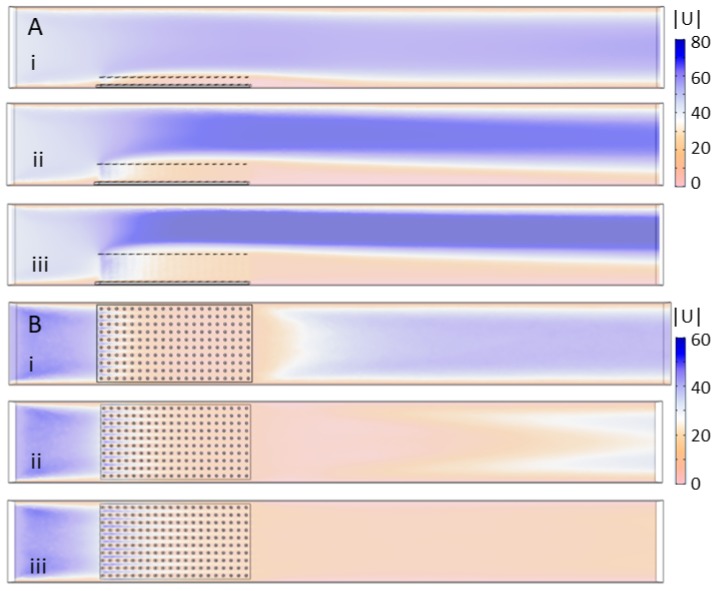
Velocity magnitude of the flow through models of varying cylinder height: (**A**) the velocity magnitude in mm/s taken in a vertical plane through the center of the tank; and (**B**) the velocity magnitude in a horizontal plane 1 cm above the bottom of the tank. The models considered include the 10 × 20 model with cylinder height equal to: (i) 1 cm; (ii) 2 cm; and (iii) 3 cm.

Figure 7 shows the velocity magnitude for the 10 × 20 20-cylinder model with height set to: (i) 1 cm; (ii) 2 cm; and (iii) 3 cm. Figure 7A shows |U| taken in a vertical plane tangential to the direction of flow through the center of the tank, and Figure 7B shows |U| in a horizontal plane 1 cm above the bottom of the tank. Figure 7A demonstrates that the bulk flow is pushed upwards and to higher velocities as the height of the model is increased. There is some non-negligible flow through the first several rows of cylinders on the upstream side, but subsequent rows are predominantly sheltered from any significant flow. The height of the wake behind the cylinders decreases in proportion to the height of the cylinders. Inspection of Figure 7B shows that variation in flow magnitude 1 cm above the bottom of the tank is small between models of different heights. In all cases, there is some flow through the first several rows of cylinders that then tapers off towards the middle of the model.

**Figure 8:**
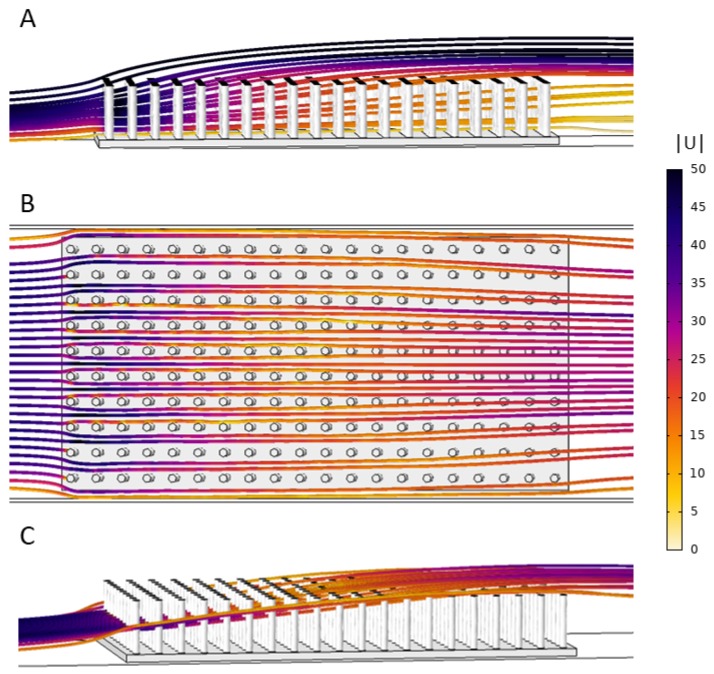
Streamlines showing the flow trajectories through the 10 × 20 model that is 2 cm in height. The color of the streamline corresponds to the velocity magnitude: (**A**) side view showing the magnitude and direction of flow through the cylinders using streamlines seeded in the vertical plane parallel to flow; (**B**) top view of streamlines that start at the midpoint of the cylinders in the *z*-direction along a horizontal line; and (**C**) the same streamlines as (**B**) in side view.

Figure 8 shows streamlines of the flow through the 10 × 20 model of cylinders that are 2 cm in height. Note that the color of the streamlines corresponds to the magnitude of the velocity. In Figure 8A, the streamlines are drawn within a vertical plane parallel to flow that is positioned in the middle of the model and between two rows of cylinders. Note that the velocity is strongest through the first few rows and the flow is directed upwards, particularly within the top half of the model. Figure 8B,C shows the flow through the same model where the streamlines are seeded along a horizontal line taken at half the cylinder height on the upstream side of the model. The flow is directed upwards, and about halfway down the model this flow is above the array of cylinders. Note that brine shrimp were injected near the bottom of the model such that the majority were advected more horizontally and within the array. The general flow pattern was similar in all models. The temporal dynamics and any eddy formations in the wakes were not resolved given the stationary equations used.

### 3.2. *Artemia* Concentration over Time

**Figure 9:**
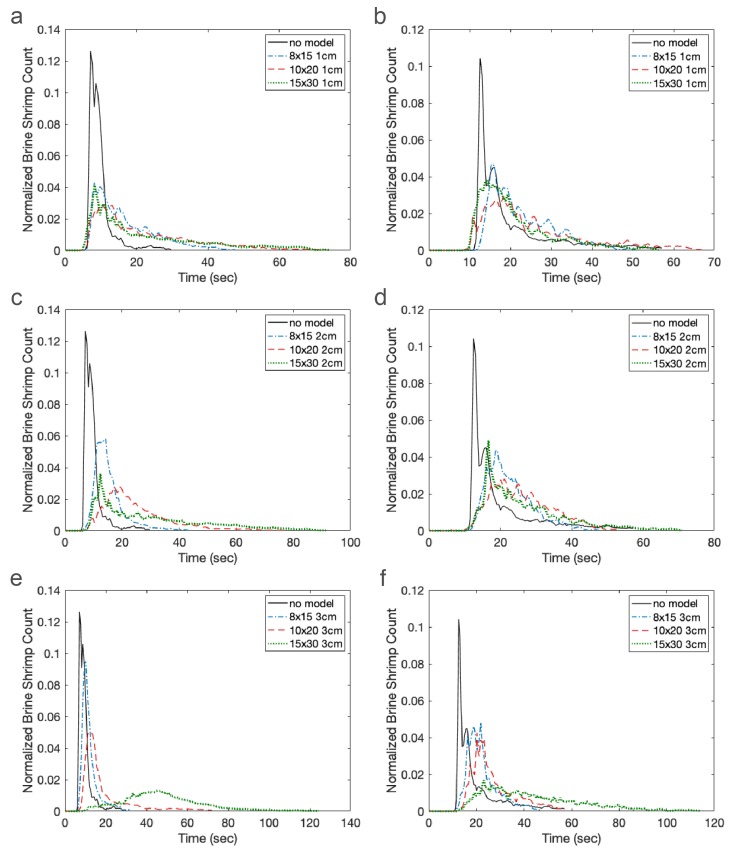
Normalized brine shrimp count over time in the green (left) and blue (right zones). Each panel shows models of the same height, namely: 1-cm cylinders (**a**,**b**); 2-cm cylinders (**c**,**d**); and 3-cm cylinders (**e**,**f**). The solid black lines show results for the no model case. Blue dotted-dashed lines, red dashed lines, and green dotted lines are for the 8 × 15, 10 × 20, and 15 × 30 models, respectively.

Figure 9 displays the number of brine shrimp in the green zone (left) and blue zone (right) as a function of time. Recall the green zone is immediately downstream of the model and the blue zone is farther downstream. The cylinder heights are set to 1 cm (Figure 9a,b), 2 cm (Figure 9c,d), and 3 cm (Figure 9e,f). In each graph, the three cylinder densities and the no model case are shown. Results were normalized by total counts of brine shrimp in the green and the blue zones. Without a model, the brine shrimp are simply advected along with the fluid with some additional random motion. This may be observed by the black line with the relatively narrow peak appearing about 8 s (green zone) and 15 s (blue zone) after the start of the experiment as well as its flat tail. Each graph also depicts the number of brine shrimp for the 8 × 15 (blue), 10 × 20 (red), and 15 × 30 (green) models. In general, increasing the density of cylinders acts to delay the presence of brine shrimp in each zone. In particular, increasing density results in a long tail such that some brine shrimp do not appear until well after the experiment has begun.

**Figure 10:**
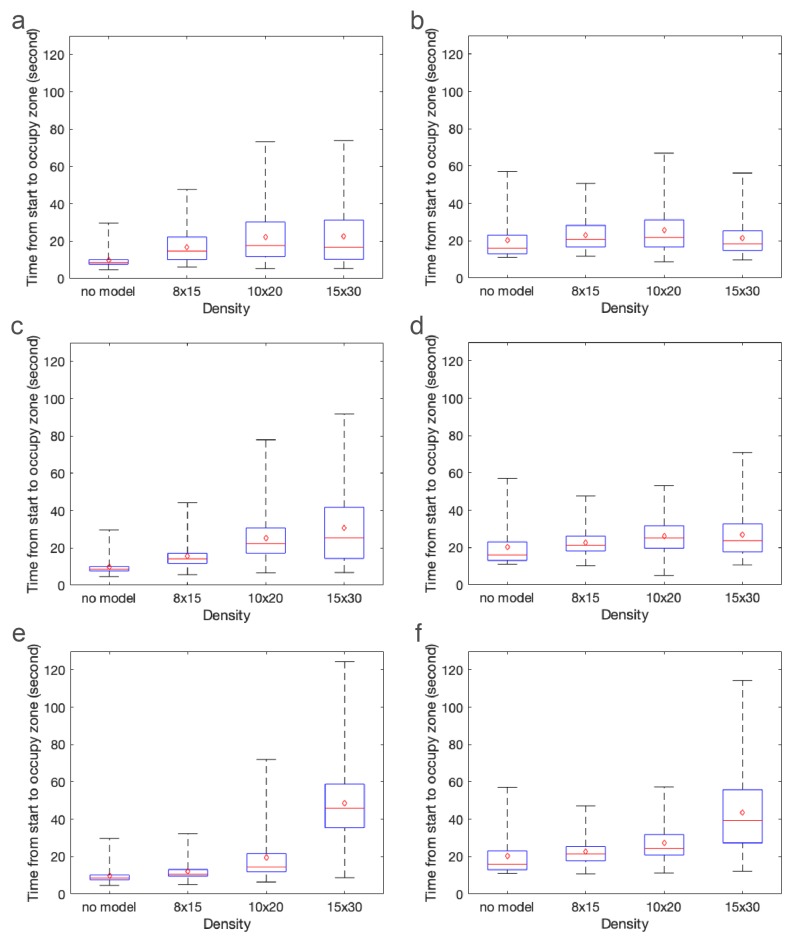
Box plots showing the mean (red dot), median (red line), upper and lower quartiles, and minimum and maximum of the distributions shown in Figure 9. Note that these statistics describe the typical times from the start of the experiment when the brine shrimp appear in the green (left) and blue (right) zones. The panels show cylinder heights set equal to: 1 cm (**a**,**b**); 2 cm (**c**,**d**); and 3 cm (**e**,**f**).

The effect of cylinder height is less clear. As found in the CFD results, the density has a much larger effect on the bulk flow through the model than does the height. To better see differences in the distributions of brine shrimp in each zone as a function of time, Figure 10 shows the time from the start of the experiment that the brine shrimp appear in the green (left zone) and blue (right zone). In other words, the mean (red dot), median (red line), upper quartile, lower quartile, minimum, and maximum of the distributions in Figure 9 are presented. Note that this is not equivalent to the arrival times presented in the next section. These plots describe the times when brine shrimp typically appear in each zone. The panels show cylinder heights set equal to 1 cm (Figure 9a,b), 2 cm (Figure 9c,d), and 3 cm (Figure 9e,f). The x-axis shows increasing density of the models.

As the density of the cylinders increases, the median, mean, and tail of the distributions of brine shrimp in the green zone increase. The trend is less apparent in the blue zone, which is farther downstream. This is possibly due to enhanced mixing in all models due to the turbulent wake. Of note is the fact that the times when the brine shrimp appear in the green and blue zones for the densest 3 cm model have a much larger mean and median and a longer tail than the other cases. This may be due to the large sheltered region downstream of the brine shrimp in this model. In general, however, the effect of height is not as straightforward, likely due to the nonlinear and non-monotonic way that bulk flow depends on height and density of a porous region in a closed channel [57].

### 3.3. Shelter Effect of Macrophyte Models

**Figure 11:**
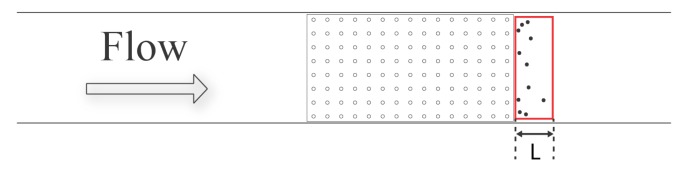
Sheltered region formed downstream of the macrophyte models. The red rectangle is a symbolic depiction of the sheltered region. This sheltered region begins at the downstream end of the macrophyte model and continuous downstream of the model. “L” is the length of the sheltered region, and it varies with the density and height of the macrophyte model.

As shown in Figure 6 and Figure 7, due to the slow down in flow past macrophyte models, some brine shrimp were able to overcome the flow velocity and remain at the bottom of the tank as depicted in Figure 11. This sheltered region of the flow can be observed immediately downstream of the model. Depending on the density and height of the model, the length of the sheltered region varied.

The effects of the model height and density on the experimentally observed dimensions of the sheltered regions were not quantified systematically, but the length of the region was the most sensitive to changes in the macrophyte model parameters. The sheltered region spanned further downstream as the height and the density of the macrophyte model increased. This sheltering effect was the most significant for the 15 × 30 3-cm model and was not observed for the empty flow tank case. Interestingly, no *Artemia* were able to stay within the macrophyte models throughout the entire duration of the experiments.

### 3.4. Agent Based Model Results

Table 1 and Table 2 show statistics for the timing of brine shrimp entering each of the green and blue zones, respectively. The models are 1 cm, 2 cm, and 3 cm in height with densities set to 8 × 15, 10 × 20, and 15 × 30. A plate case (no model) is also shown. In each case, 10,000 agents were simulated with a starting position 1 mm behind the center of the model and 3 mm from the bottom of the simulated tank. From this point, each agent underwent unbiased Brownian motion with a variance of 2.5 mm${}^{2}$/s while being advected by the flow. This number was based on the diffusion constant *D* for brine shrimp reported in Figure 5 and Table 2 (video data) in [39] (D≈0.025 cm${}^{2}$/s). Variance is given as 2D, but we halved the value to account for fully 3D movement as opposed to the largely 2D domain considered in [39]. Each simulation was run until all agents had entered both zones.

**Figure 12:**
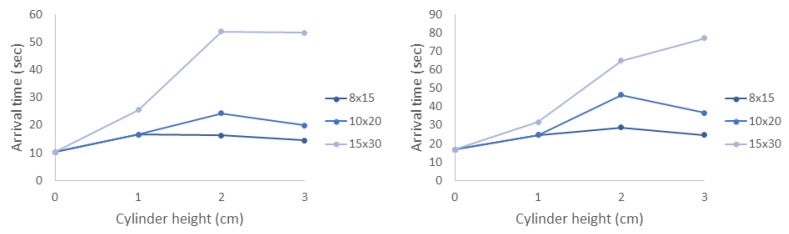
The average arrival time vs. cylinder height for agents in the simulations into: the green zone (**left**); and blue zone (**right**).

In all cases, the average arrival time increased with the density of the cylinders. Furthermore, the standard deviation of the arrival times was much larger for the densest 15 × 30 model. The relationship between arrival time and cylinder height was less obvious. This effect was also observed in the experiments, and it is worth noting that the flow velocities near where the nauplii were injected did not change appreciably with height. These trends are easily observed in Figure 12, which shows the average arrival time as a function of cylinder height. For any given cylinder height, the average arrival time increases with model density.

Table 3 and Table 4 show statistics for the timing of brine shrimp entering each of the green and blue zones, respectively, for a 10 × 20 2-cm model with varying levels of brine shrimp effective diffusivity. Note that a diffusivity of zero (D=0) corresponds to the movement of passive tracers, and increasing *D* corresponds to faster swimming speeds. As before, in each case, 10,000 agents were simulated with a starting position 1 mm behind the center of the model and 3 mm from the bottom of the simulated tank. Note that the standard deviation of the arrival times for the case when D=0 is zero as all of the agents are released together and travel together.

Interestingly, the mean and median arrival times initially increase with increasing diffusivity before decreasing. At low diffusivities, some of the agents move to very slow moving regions of the flow and stay there for some time. As the diffusivity increases, most agents enter regions of faster moving flow in a relatively short amount of time and are swept downstream. This result suggests that there may be an optimal effective diffusivity that maximizes arrival times and increases the duration of time that the plankton remain in the macrophyte layer.

## 4. Discussion

In this paper, we develop an open source agent-based code for simulating plankton movement in complex flow fields. This framework was applied to a relatively simple case of brine shrimp moving through an idealized model of a macrophyte bed at the scale of tens of centimeters. Using this simple test case, we found that: (1) arrival times are largely affected by the density of the macrophyte model; (2) the effect of macrophyte height on arrival times is not straightforward; and (3) downstream arrival times are a non-monotonic function of the effective diffusivity of the agents. Predictive models of the dispersal of plankton whose trajectories are significantly determined by both the local fluid flow and their own behavior are important for many conservation applications. For example, high fidelity dispersal models of invasive species such as some bivalves [58,59], starfish [60,61] and copepods, for example, *Oithona davisae* [58], could focus removal efforts and inform the timing and location of the release of biocontrol agents. Models of the dispersal of the biocontrol agents themselves could allow more effective release of these organisms. In terms of the role of macrophytes and other protective structures in sheltering plankton, predictive models could inform the best placement and geometry of artificial reefs and could identify key locations where vegetation and reefs should be protected.

Work to leverage existing and further develop state-of-the-art numerical methods and mathematical analyses to reveal how organism swimming performance, behavior, and environment conditions alter dispersal and subsequent distributions of plankton have applications to multiple areas of biological oceanography and conservation. Using theoretically and experimentally determined estimates of thrust and mathematical models of behavior (including preferred dispersal conditions), scientists could predict and validate dispersal distributions across a variety of landscapes. Significant consideration needs to be given to the mathematical challenges in describing the dispersal of tiny organisms, including: (i) modeling techniques to connect dynamics at various spatial and temporal scales; (ii) data collection protocols and data accessibility; (iii) agent-based models that incorporate animal behavior and swimming trajectories; and (iv) code generation that is well-documented and generally applicable for both education and research. The primary goal of this study was to document such an approach for a model organism in an idealized setting.

The results of this paper clearly show that both the average time to arrive in each zone and the variance depend strongly on the density of the macrophytes. This is primarily due to the strong nonlinear effect that cylinder density has on local flow speed as well as the region of flow that is sheltered downstream of the model. Cylinder height has a weaker effect on arrival times, likely due to the fact that the brine shrimp are injected near the bottom of the tank where differences in flow speeds are negligible. One exception to this is that the densest and tallest model greatly increased the time that brine shrimp arrived or appeared in the zones in both the experiments and the simulations. This is likely due to the large, sheltered region downstream of the model.

These results clearly demonstrate that the density of macrophytes, natural and artificial reefs, and other structures has a strong effect on plankton retention time. Since there is a strong nonlinear transition where such structures act as either nearly impermeable regions or porous layers with significant leakiness [36,62], it is critical that sheltering structures be above this leaky-to-solid threshold. Furthermore, dense, tall structures may create slow flow regions downstream that could also shelter plankton.

Our results also suggest areas for future research. Our numerical simulations of the flow fields ignored unsteady effects that could either increase the spatial variance of the brine shrimp due to fluctuations in the velocity field or condense the spatial distribution given the effect of separatrices in the flow. It was also assumed that the brine shrimp move in a way that is well described by an unbiased random walk. It is known that nauplii use phototaxis and may avoid regions of strong flow or high shear. Any bias in the movement to swim together or apart would also alter their distributions. These higher-order effects underscore the need for more complex models that consider both behavior and temporally varying flow fields.

We introduced an open source, agent-based framework called *Planktos* to quantify emergent spatial and temporal patterns of plankton and other small organisms in complex flows based on individual-level behavioral rules. The unique feature of this open source platform is that the movement of agents is easily coupled to flow. This feature makes the software particularly suitable for modeling the movement of marine, aquatic, and aerial plankton across the scales of centimeters to tens of meters. Accordingly, the code should find immediate use in applications focused on resolving the movement of plankton within complex flow fields such as water flow through macrophyte beds and reefs. Massive agents may also be easily introduced to consider the dispersal of aerial plankton (small insects, spiders, etc.) due to winds through a crop field or forest patch.

## Figures and Tables

**Table 1:** Statistics for the timing of brine shrimp agents entering the green zone by model. All values are given in units of seconds, and both skewness and kurtosis are calculated as Pearson standardized moments.

	Mean	Median	Mode	Std	Skewness	Kurtosis
plate	10.26556	7.60	4.8	6.644776	1.577448	5.648092
8 × 15_1 cm	16.60715	11.80	7.1	12.855364	1.867912	7.226498
8 × 15_2 cm	16.34799	12.80	7.1	10.679333	1.703749	6.641543
8 × 15_3 cm	14.50401	11.40	5.4	9.753728	1.685753	6.397291
10 × 20_1 cm	16.60729	11.80	7.1	12.855351	1.867895	7.226457
10 × 20_2 cm	24.37854	20.55	15.9	12.792898	1.755368	7.025288
10 × 20_3 cm	20.02696	16.80	11.0	10.779727	1.657012	6.375421
15 × 30_1 cm	25.48717	15.20	7.5	29.562239	3.637440	21.971364
15 × 30_2 cm	53.94625	35.00	20.3	51.528729	2.332308	9.424739
15 × 30_3 cm	53.52796	51.20	48.6	23.670932	0.971755	4.696066

**Table 2:** Statistics for the timing of brine shrimp agents entering the blue zone by model. All values are given in units of seconds, and both skewness and kurtosis are calculated as Pearson standardized moments.

	Mean	Median	Mode	Std	Skewness	Kurtosis
plate	16.73456	14.1	8.5	8.821590	1.266366	4.496077
8 × 15_1 cm	24.60723	19.4	12.3	15.809911	1.596273	6.157659
8 × 15_2 cm	28.81300	25.1	21.7	13.554596	1.344732	5.116872
8 × 15_3 cm	24.74002	21.7	16.3	11.189394	1.478259	5.706537
10 × 20_1 cm	24.60797	19.4	12.3	15.810498	1.596170	6.157190
10 × 20_2 cm	46.45774	38.0	29.0	27.945304	1.706768	6.729241
10 × 20_3 cm	36.81893	32.8	25.4	14.341388	1.438209	5.511163
15 × 30_1 cm	31.69173	20.6	12.3	31.655163	3.339417	19.205411
15 × 30_2 cm	65.00588	42.2	23.5	62.218139	2.383948	9.495929
15 × 30_3 cm	77.31394	67.3	39.9	48.245441	2.082177	10.752551

**Table 3:** Statistics for the timing of brine shrimp agents entering the green zone by diffusivity. All values are given in units of seconds, and both skewness and kurtosis are calculated as Pearson standardized moments.

2·D (mm${}^{2}$/s)	Mean	Median	Mode	Std	Skewness	Kurtosis
0	13.50000	13.50	13.5	0.000000	0.000000	0.000000
0.25	34.90953	21.40	11.5	36.072997	2.916802	13.992966
0.5	33.21686	23.90	11.7	27.674093	2.490382	11.650339
0.75	31.17568	23.60	12.2	23.050775	2.357897	10.953085
1	29.57997	23.20	13.9	19.933192	2.237405	10.726133
2.5	24.37854	20.55	15.9	12.792898	1.755368	7.025288
5	20.75572	18.30	15.8	9.750290	1.376773	5.397198
7.5	18.78547	17.00	13.9	8.774293	1.223732	5.023428
10	17.39927	15.90	16.0	8.201403	1.088226	4.535014
25	13.30244	11.90	7.3	6.887721	0.957236	3.731665

**Table 4:** Statistics for the timing of brine shrimp agents entering the blue zone by diffusivity. All values are given in units of seconds, and both skewness and kurtosis are calculated as Pearson standardized moments.

2·D (mm${}^{2}$/s)	Mean	Median	Mode	Std	Skewness	Kurtosis
0	69.30000	69.3	69.3	0.000000	0.000000	0.000000
0.25	85.77890	59.9	42.1	65.286745	2.185369	8.832309
0.5	74.05316	54.6	35.8	52.298372	2.005044	7.897292
0.75	66.46952	50.3	33.8	45.059692	2.005264	8.396994
1	61.38943	47.3	34.7	40.119159	1.877701	7.387855
2.5	46.45774	38.0	29.0	27.945304	1.706768	6.729241
5	37.13526	31.4	17.5	21.720836	1.463269	5.702837
7.5	32.43831	27.5	21.2	19.078510	1.347951	5.084803
10	29.31992	24.8	15.1	17.390467	1.294047	4.795384
25	21.03663	17.3	8.8	12.828173	1.295601	4.659608

## References

[1] Havenhand J.N., McEdward L. (1995). Evolutionary ecology of larval types. Ecology of Marine Invertebrate Larvae.

[2] Fenchel T. (1988). Marine plankton food chains. Annu. Rev. Ecol. Syst..

[3] Sarkar R.R., Chattopadhayay J. (2003). Occurrence of planktonic blooms under environmental fluctuations and its possible control mechanism—Mathematical models and experimental observations. J. Theor. Biol..

[4] Hallegraeff G.M. (1993). A review of harmful algal blooms and their apparent global increase. Phycologia.

[5] Levin L.A. (2006). Recent progress in understanding larval dispersal: New directions and digressions. Integr. Comp. Biol..

[6] Cianelli D., D’Alelio D., Uttieri M., Sarno D., Zingone A., Zambianchi E., d’Alcala M.R. (2017). Disentangling physical and biological drivers of phytoplankton dynamics in a coastal system. Sci. Rep..

[7] Lewis D.M., Brereton A., Siddons J.T. (2017). A large eddy simulation study of the formation of deep chlorophyll/ biological maxima in unstratified mixed layers: The roles of turbulent mixing and predation pressure. Limnol. Oceanogr..

[8] Pena M.A., Masson D., Callendar W. (2016). Annual plankton dynamics in a coupled physical-biological model of the Strait of Georgia, British Columbia. Prog. Oceanogr..

[9] Papworth D.J., Marini S., Conversi A. (2016). A novel, unbiased analysis approach for investigating population dynamics: A case study on Calanus finmarchicus and its decline in the North Sea. PLoS ONE.

[10] Schlacher T.A., Wooldridge T.H. (1995). Small-scale distribution and variability of demersal zooplankton in a shallow, temperate estuary: Tidal and depth effects on species-specific heterogeneity. Cah. Biol. Mar..

[11] Lundquist C.J., Pilditch C.A., Cummings V.J. (2004). Behaviour controls post-settlement dispersal by the juvenile bivalves Austrovenus stutchburyi and Macomona liliana. J. Exp. Mar. Biol. Ecol..

[12] Shurin J.B., Cottenie K., Hillebrand H. (2009). Spatial autocorrelation and dispersal limitation in freshwater organisms. Oecologia.

[13] McKenna J.E., Chalupnicki M.A. (2011). A heuristic simulation model of Lake Ontario circulation and mass balance transport. J. Freshw. Ecol..

[14] Pepin P., Guoqi H., Head E.J. (2013). Modelling the disperal of Calanus finmarchicus on the Newfoundland Shelf: Implications for the analysis of population dynamics from a high frequency monitoring site. Fish. Oceanogr..

[15] Hill A.E. (1991). Advection-diffusion-mortality solutions for investigating pelagic larval dispersal. Mar. Ecol. Prog. Ser..

[16] Lefebvre A., Ellien C., Davoult D., Thiébaut E., Salomon J. (2003). Pelagic dispersal of the brittle-star Ophiothrix fragilis larvae in a megatidal area (English Channel, France) examined using an advection/diffusion model. Estuar. Coast. Shelf Sci..

[17] Shanks A.L., Grantham B.A., Carr M.H. (2003). Propagule dispersal distance and the size and spacing of marine reserves. Ecol. Appl..

[18] Gibson R. (2003). Go with the flow: Tidal migration in marine animals. Hydrobiologia.

[19] McManus M., Woodson C. (2012). Plankton distribution and ocean dispersal. J. Exp. Biol..

[20] Clay T.W., Grunbaum D. (2010). Morphology-flow interactions lead to stage-selective vertical transport of larval sand dollars in shear flow. J. Exp. Biol..

[21] Desai N., Ardekani A.M. (2017). Modeling of active swimmer suspensions and their interactions with the environment. Soft Matter.

[22] Nepf H. (2012). Flow and Transport in Regions with Aquatic Vegetation. Annu. Rev. Fluid Mech..

[23] Belcher S.E., Harman I.N., Finnigan J.J. (2012). The wind in the willows: Flows in forest canopies in complex terrain. Ann. Rev. Fluid Mech..

[24] Jadhav R.S., Buchberger S.H. (1995). Effects of vegetation on flow through free surface wetlands. Ecol. Eng..

[25] Raupach M.R., Finnigan J.J., Brunet Y. (1996). Coherent eddies and turbulence in vegetation canopies: The mixing-layer analogy. Bound.-Layer Meteorol..

[26] Poggi D., Katul G.G., Albertson J.D. (2004). A note on the contribution of dispersive fluxes to momentum transfer within canopies. Bound.-Layer Meteorol..

[27] Bos A.R., Bouma T.J., de Kort G.L.J., van Katwijk M.M. (2007). Ecosystem engineering by annual intertidal seagrass beds: Sediment accretion and modification. Estuar. Coast. Shelf Sci..

[28] Temmerman S., Bouma T.J., Govers G., Wang Z.B., Vries M.B.D., Herman P.M.J. (2005). Impact of vegetation on flow routing and sedimentation patterns: Three-dimensional modeling for a tidal marsh. J. Geophys. Res..

[29] Pasour V.B., Ellner S.P. (2010). Computational and Analytic Perspectives on the Drift Paradox. SIAM J. Appl. Dyn. Syst..

[30] Gambi M.C., Nowell A.R., Jumars P.A. (1990). Flume observations on flow dynamics in Zostera marina (eelgrass) beds. Mar. Ecol. Prog. Ser..

[31] Jarvela J. (2002). Flow resistance of flexible and stiff vegetation: A flume study with natural plants. J. Hydrol..

[32] Ackerman J.D., Okubo A. (1993). Reduced mixing in a marine macrophyte canopy. Funct. Ecol..

[33] Finnigan J. (2000). Turbulence in plant canopies. Annu. Rev. Fluid Mech..

[34] Raupach M.R., Shaw R.H. (1982). Averaging procedures for flow within vegetative canopies. Bound.-Layer Meteorol..

[35] Wilson N.R., Shaw R.H. (1977). A higher order closure model for canopy flow. J. Appl. Meteorol..

[36] Strickland C., Miller L., Santhanakrishnan A., Hamlet C., Battista N.A., Pasour V. (2017). Three-Dimensional Low Reynolds Number Flows near Biological Filtering and Protective Layers. Fluids.

[37] Miller L.A., Santhanakrishnan A., Jones S., Hamlet C.L., Mertens K., Zhu L. (2012). Reconfiguration and the reduction of vortex-induced vibrations in broad leaves. J. Exp. Biol..

[38] Maltese A., Cox E., Folkard A.M., Ciraolo G. (2007). Laboratory measurements of flow and turbulence in discontinuous distributions of ligulate seagrass. J. Hydraul. Eng..

[39] Kohler B.R., Swank R.J., Haefner J.W., Powell J.A. (2010). Leading Students to Investigate Diffusion as a Model of Brine Shrimp Movement. Bull. Math. Biol..

[40] Railsback S.F., Grimm V. (2012). Agent-Based and Individual-Based Modeling: A Practical Introduction.

[41] Huth A., Drechsler M., Köhler P. (2004). Multicriteria evaluation of simulated logging scenarios in a tropical rain forest. J. Environ. Manag..

[42] D’Orsogna M.R., Chuang Y.L., Bertozzi A.L., Chayes L.S. (2006). Self-propelled particles with soft-core interactions: Patterns, stability, and collapse. Phys. Rev. Lett..

[43] Liedloff A.C., Cook G.D. (2007). Modelling the effects of rainfall variability and fire on tree populations in an Australian tropical savanna with the FLAMES simulation model. Ecol. Model..

[44] Schulze J., Müller B., Groeneveld J., Grimm V. (2017). Agent-Based Modelling of Social-Ecological Systems: Achievements, Challenges, and a Way Forward. J. Artif. Soc. Soc. Simul..

[45] Netlogo References. https://ccl.northwestern.edu/netlogo/references.shtml.

[46] Topaz C.M., Bernoff A.J., Logan S., Toolson W. (2008). A model for rolling swarms of locusts. Eur. Phys. J. Spec. Top..

[47] Nilsen C., Paige J., Warner O., Mayhew B., Sutley R., Lam M., Bernoff A.J., Topaz C.M. (2013). Social Aggregation in Pea Aphids: Experiment and Random Walk Modeling. PLoS ONE.

[48] Railsback S.F., Lytinen S.L., Jackson S.K. (2006). Agent-based Simulation Platforms: Review and Development Recommendations. SIMULATION.

[49] Purcell E.M. (1977). Life at low Reynolds number. Am. J. Phys..

[50] Williams T.A. (1994). Locomotion in Developing Artemia Larvae: Mechanical Analysis of Antennal Propulsors Based on Large-Scale Physical Models. Biol. Bull..

[51] Williams T.A. (1994). A Model of Rowing Propulsion and the Ontogeny of Locomotion in Artemia Larvae. Biol. Bull..

[52] Vogel S., LaBarbera M. (1978). Simple Flow Tanks for Research and Teaching. BioScience.

[53] Raffel M., Willert C., Kompenhans J. (1998). Particle Image Velocimetry: A Practical Guide.

[54] Willert C.E., Gharib M. (1991). Digital particle image velocimetry. Exp. Fluids.

[55] MATLAB (2017). Version 9.3.0.713579 (R2017b).

[56] Strickland C. (2018). Planktos Agent-Based Modeling Framework. https://github.com/mountaindust/Planktos.

[57] Leiderman K.M., Miller L.A., Fogelson A.L. (2008). The effects of spatial inhomogeneities on flow through the endothelial surface layer. J. Theor. Biol..

[58] Altukhov D.A., Gubanova A.D., Mukhanov V.S. (2014). New invasive copepod Oithona davisae Ferrari and Orsi, 1984: Seasonal dynamics in Sevastopol Bay and expansion along the Black Sea coasts. Mar. Ecol..

[59] Hoyer A.B., Wittmann M.E., Chandra S., Schladow S.G., Rueda F.J. (2014). A 3D individual-based aquatic transport model for the assessment of the potential dispersal of planktonic larvae of an invasive bivalve. J. Environ. Manag..

[60] Scandol J.P., James M.K. (1992). Hydrodynamics and larval dispersal: A population model of Acanthaster planci on the Great Barrier Reef. Mar. Freshw. Res..

[61] Van der Laan J., Bradbury R. (1990). Futures for the Great Barrier Reef ecosystem. Math. Comput. Model..

[62] Cheer A., Koehl M. (1987). Paddles and rakes: Fluid flow through bristled appendages of small organisms. J. Theor. Biol..

